# Hepatitis C in Egypt – past, present, and future

**DOI:** 10.2147/IJGM.S119301

**Published:** 2016-12-20

**Authors:** Ahmed Elgharably, Asmaa I Gomaa, Mary ME Crossey, Peter J Norsworthy, Imam Waked, Simon D Taylor-Robinson

**Affiliations:** 1 Division of Digestive Health, Department of Surgery and Cancer, St Mary’s Hospital, Imperial College London, London, UK; 2 National Liver Institute, Menoufiya University, Shebeen El Kom, Egypt

**Keywords:** hepatitis C, Egypt, schistosomiasis

## Abstract

Hepatitis C viral infection is endemic in Egypt with the highest prevalence rate in the world. It is widely accepted that the implementation of mass population antischistosomal treatment involving administration of tartar emetic injections (from 1950s to 1980s) led to widespread infection. What is less well known, however, is that these schemes were implemented by the Egyptian Ministry of Health on the advice of the World Health Organization. There has been a spectrum of treatments to target the public health disaster represented by the hepatitis C problem in Egypt: from the use of PEGylated interferon to the recent use of direct acting antiviral drugs. Some new treatments have shown >90% efficacy. However, cost is a key barrier to access these new medicines. This is coupled with a growing population, limited resources, and a lack of infection control practices which means Egypt still faces significant disease control issues today.

## Introduction

This article aims to put the hepatitis C viral (HCV) epidemic in Egypt into a historical and cultural context, given that it arose in part from public health measures encouraged by Egyptian health authorities and World Health Organization (WHO) officials in a period spanning over three decades in the latter half of the last century. The long natural history of the condition has meant that the country is still grappling with the effects of these policies to the present day. However, new treatments for HCV with direct-acting antiviral agents allow, for the first time, the prospect of future eradication of the condition. This article aims to highlight these prospects against the economic costs of such treatment and the steps that have been taken to obtain the widest possible treatment coverage.

HCV, and its long-term resultant consequences, is a major endemic medical health problem in Egypt. Having taken a representative sample of the country, from both urban and rural areas, an Egyptian demographic health survey conducted in 2008 concluded that 14.7% of the population have been infected, making this the highest prevalence in any population in the world.^1^–^3^ In the Nile Delta and Upper Egypt, infection rates can be much higher at around 26% and 28%, respectively.^4^ With incidence rates between 2 and 6 per 1000 every year, this leads to an estimated 170,000 new cases every year to add to the 11.5 million patients suffering from the disease.^4^

More recent epidemiological modeling studies conducted to assess the HCV disease burden have suggested a more conservative estimate of 7.3% of the population with viremic HCV in 2013.^5^,^6^ This is mainly due to the mortality in the older age groups who have the highest prevalence of infection.

HCV infection occurs through blood contact.^2^ Apart from the usual modes of transmission, such as intravenous drug usage, the main risk factors for transmission in Egypt historically have included the now archaic parenteral antischistosomal therapy, shared or reused needles, poorly sterilized surgical or dental equipment, and blood transfusions.^7^ In the past, it was primarily the use of widespread tartar emetic injections, which were used to treat schistosomiasis in Egypt in the 1950s to the early 1980s, which laid the foundation for the HCV epidemic currently seen. Since it can take up to 20–30 years for HCV infection to become clinically evident, there has been a lag phase of several decades before the problem became apparent. While currently, Egypt is still seeing a few new cases of hepatitis C-related liver disease presenting from the initial antischistosomal campaign, with some patients displaying a lag phase of 40 years before clinical presentation, in practice, poor infection control and equipment sterilization procedures used in medical and dental settings also led and continue to lead to iatrogenic HCV infections to the present day, which further stimulate the spread of the disease and continue to fuel the current epidemic.^2^

Although around 30% of patients may clear the virus spontaneously,^8^ the main health burden occurs from the majority of patients who develop chronic HCV. In this patient population, cirrhosis may develop within 20 years of infection.^8^ With hepatic decompensation and hepatocellular carcinoma, these long-term consequences have put further strain on resources in an already overstretched Egyptian healthcare system.^4^,^9^

### What is HCV?

**Figure 1:**
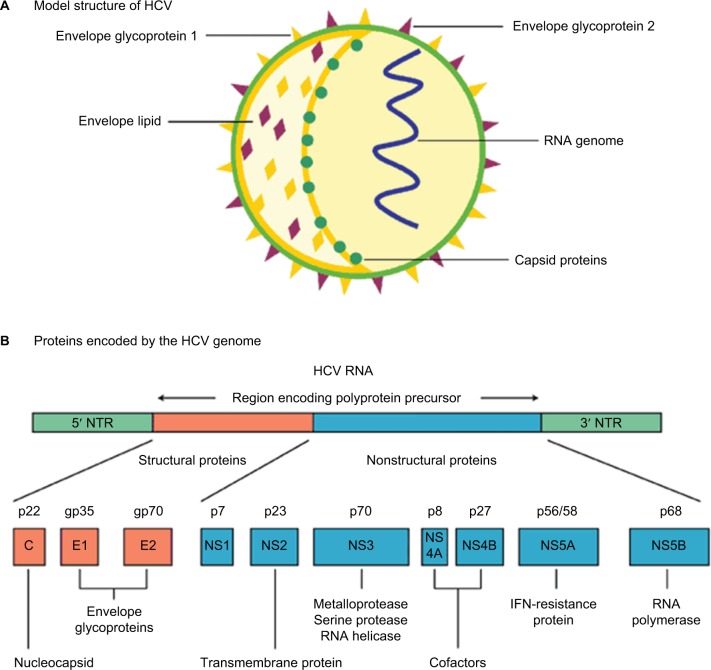
HCV: model structure and genome organization. **Notes:** (**A**) Model structure of HCV. (**B**) Proteins encoded by the HCV genome. Expert review in Molecular Medicine © 2003 Cambridge University Press.^39^ **Abbreviations:** HCV, hepatitis C viral; IFN, interferon; NTR, nontranslated RNA.

HCV is a hepatotropic RNA virus of the genus *Hepacivirus* in the Flaviviridae family.^10^–^12^ The virus exists as an enveloped, positive-stranded RNA virus which is ~50 nm in size (Figure 1). The HCV RNA strand is made up of ~9600 nucleotide bases and is covered by an icosahedral nucleocapsid which is further surrounded by a lipid bilayer and glycoproteins. HCV is grouped into 6 major genotypes that exhibit at least 30% variation in nucleotide sequence from one another.^10^–^12^ This genetic variation within the population is a powerful selection mechanism for resistance to both medicinal drugs and evasion of the immune system.^10^,^12^ The most common HCV RNA genotype in Egypt is genotype 4, representing >85% of all HCV cases in Egypt.

### History of the infection

With HCV seroprevalence at up to 40% in some areas of Egypt,^13^–^16^ based on blood-bank surveys, it is obvious that HCV is a huge public health issue within the country.^17^–^19^ A particular focus is placed on the Nile delta region which holds the greatest rates of infection and was, historically, the main focus for schistosomiasis.^2^ The primary two schistosome species in Egypt are *Schistosoma mansoni* and *Schistosoma haematobium*.^20^,^21^ Until the HCV epidemic became apparent, schistosomiasis was the most important public health problem in Egypt with *S. manson*i being the primary cause of liver disease within Egypt historically.^22^

In 1918, JB Christopherson made the discovery that injections with the antimony salt, tartar emetic, could induce a cure.^2^,^20^ Egypt, at the time, had the greatest schistosomiasis burden in the world, and mass treatment of the parasite was introduced via primary health care services.^20^

From the 1950s to the 1980s, community-wide mass antischistosomal therapy was introduced by the Egyptian Ministry of Health with the advice and support of the WHO.^22^–^24^ At the time, tartar emetic injections were the standard treatment. They were injected intravenously, unlike some other now-archaic antischistosomal drugs that were injected intramuscularly. Over 2 million injections were given annually to an average of 250,000 patients, meaning over the 18 years of treatment, 36 million injections were administered.^2^,^20^ Each patient was supposed to have a series of injections with the average number of injections per patient being nine in the 1960s, which then dropped to six after 1975.^20^,^21^

There are three main causes for the transmission of HCV, as well as other blood-borne diseases in this mass treatment scheme.^20^ First, patients were exposed to multiple injections over the time period which increased the likelihood of pathogen transmission. Second, sterilization techniques were extremely poor, which led to high frequency of HCV transmission, a virus that was not known to medical science until the 1990s. Finally, the mass scale of the antischistosomal eradication campaign led to widespread mistakes, including reuse of equipment, which was something not considered important until the advent of the HIV epidemic in the early- to mid-1980s.^2^

To add to the danger of the campaign, “acute clinical symptoms are not present in about 80% of HCV infections.”^20^ This means infection spread rapidly and would go largely unnoticed. These campaigns are hypothesized to have led to the “high HCV seroprevalency rates currently observed in the Nile delta”.^2^,^13^,^16^,^23^,^24^, This hypothesis is further supported by the clustering effect observed between HCV infections in households with patients who received parenteral treatment for schistosomiasis.^23^

Toward the end of the campaign in the 1970s, oral drugs to treat schistosomiasis were developed, including the oral agent, praziquantel, which slowly replaced the tartar emetic injections as the gold standard of treatment.^20^,^22^

### How HCV affects the liver

Hepatitis C viral infection is largely asymptomatic with little visible symptoms in its acute infection stage. It is only when a patient has been harboring the disease for anywhere between 20–40 years and therefore has a chronic HCV infection when noticeable symptoms or signs will occur. HCV is a significant “precursor” for fibrosis, cirrhosis, and ultimately, hepatocellular carcinoma, but it is important to understand this is only in long-term, chronic cases.^20^,^25^ In Egypt, up to 85% of HCV infections persist for life, leading to chronic hepatitis.^20^,^27^ The major cause of death is primarily associated with cirrhosis in the liver as well as other conditions including liver failure, hematemesis from esophageal varices, hepatic encephalopathy and hepatocellular carcinoma.^20^,^28^ HCV may complicate the course of schistosomiasis and vice versa with a perhaps synergistic effect. A long-term study showed that complications occurred at a much faster rate in those with coinfection with around 48% having cirrhosis, compared with 15% in those who had HCV alone and 0% in the group with schistosomiasis alone.^20^

### Previous and current treatments

Interferon treatment was incredibly costly especially for a largely poor patient population in Egypt, which meant that access to treatment was sporadic.^20^,^28^ Focus for HCV treatment has therefore switched to promoting the research and development of direct acting antiviral (DAA) drugs which present an oral, interferon-free treatment option.^28^,^29^ The drugs are specific to the HCV particle and aim to inhibit viral RNA replication by attacking some of the several enzymes involved in the RNA replication process, thereby inhibiting viral replication and causing viral eradication.^29^ This represents a novel and less invasive treatment scheme which has proven revolutionary in tackling the HCV epidemic in Egypt.

### Efficacy of treatment with sofosbuvir and ribavirin

In a study of the DAA combination, sofosbuvir and ribavirin, pan-genotypic clinical efficacy in HCV genotypes 1–6 was demonstrated.^30^,^31^ Another stage 3 clinical trial involving giving patients sofosbuvir in conjunction with ribavirin and PEGylated alpha interferon for a 12-week treatment period showed a sustained virological response in 27 out of 28 treatment-naïve patients with HCV genotype 4 (96% efficacy).^30^ The efficacy of treatment has been profound to the extent that the European Association for the Study of the Liver and the WHO have recommended either a course of sofosbuvir, ribavirin and PEGylated alpha-interferon for a 12-week treatment period or 24 weeks of ribavirin in conjunction with sofosbuvir as an interferon-free treatment regime.^32^–^34^ Other direct acting antiviral treatments include simeprevir, daclatasvir, a ledipasvir–sofosbuvir combination tablet, and a paritaprevir–ombitasvir combination tablet.

### Accessibility of treatment

This study highlighted that while HCV is a prevalent and difficult medical problem in Egypt, there are existing drug regimens which have a proven efficacy in tackling the disease. One of the biggest problems facing the Egyptian population, however, comes in access to these drug treatments – especially when considering that the HCV epidemic in Egypt is one of a largely socioeconomic nature.^1^,^35^ The condition is most prevalent within rural communities compared with urban communities (12% and 7% prevalence rates, respectively).^1^ As well as this, HCV varies with wealth too, having a 12% prevalence in the lowest quartile compared with 7% in the upper quartile of the population.^1^ It is therefore the case that the poorest and least educated in society suffer the problematic effects of this epidemic which poses challenges both in access and affordability of treatment but also in diagnosing the true extent of the problem due to high illiteracy rates and low HCV awareness levels.^36^

Between 2008 and 2012, the Egyptian National Committee for the Control of Viral Hepatitis (established in 2006) aimed to develop a strategy to control viral hepatitis. This involved investing US$80 million each year with the aim of treating 20% of the HCV patients in Egypt by the end of 2012.^36^,^37^ The scheme would subsidize the preferred treatment at the time – which was PEGylated alpha interferon with ribavirin. By the end of 2011, around 2.8% of patients had benefited from HCV treatment with only 114,000 achieving a sustained virological response (1.67%). The reasons for this low cure rate are multifactorial, but principally include poor patient supervision and poor patient compliance with, or adherence to a 6–12 month interferon-based regimen that had significant side effects including lethargy, depression, and “flu-like” symptoms. When this scheme was a national policy, only patients with relatively higher chances of cure had access to treatment.^36^,^37^ At the time the Egyptian Ministry of Health, also, put too much emphasis on treatment, compared with infection control and education which with hindsight proved to be a key failure of the plan.^19^

The Egyptian Ministry of Health has since proposed a new national strategy to control the HCV epidemic in Egypt with a greater capital fund and with support from the WHO as well as other institutes.^19^ This scheme was entitled “The Plan of Action for the Prevention, Care and Treatment of Viral Hepatitis 2014–2018” and promoted sofosbuvir (Sovaldi™, Gilead Sciences, San Francisco, USA) as its primary treatment.^36^ The scheme aims to treat 300,000 patients annually and the cost of treatment would be distributed between the Egyptian Ministry of Health (38%), the Egyptian Health Insurance Organization (51%), private payments (3%), and finally cash payments from patients (8%).^14^,^36^

Sovaldi retails for US$84,000 for a 12-week course making it a staggering US$1000 per pill.^36^ This makes it out of reach for the Egyptian HCV patient population. Negotiations between the Egyptian government in the form of the “National Committee for the Control of Viral Hepatitis” and Gilead Sciences resulted in a reduced cost of US$300 per box of Sovaldi which would supply 1 month of treatment.^36^ Headlines portrayed this as a 99% discount on the original price of the treatment. However, the reality is that only patients treated under the governmental treatment scheme are currently able to access this discounted price.^38^ Gilead registered the same product on the private pharmaceutical market for EGP 14,900 per box.^36^ This makes the private cost of treatment around six times the government price. Hence, due to the limited availability and reach of the governmental scheme, patients are still faced with unaffordable drug treatment. However, there is hope that the subsidized scheme will be extended in the future as part of Egyptian Government policy and/or Gilead corporate social responsibility. Such negotiations are ongoing at the time of writing. This would then offer the real possibility of HCV eradication in Egypt for the first time.

## Conclusion/Discussion

### Alternative treatments and future steps

It is clear that there is evidence of some good foundations for tackling the widespread prevalence of HCV in Egypt. At the time of writing, the development of effective DAAs has shown up to 90% efficacy against HCV genotype 4, and such treatment has been able to produce excellent SVR results in the Egyptian context. There is less ignorance and lack of awareness of the need to properly sterilize equipment and medical instruments than there has been in the past, although public awareness campaigns still need to be conducted, including to allied health care professionals. From a historical perspective, the Egyptian population has become much more aware of the threats associated with swimming and utilizing canal water which has led to a decrease in incidence rates of schistosomiasis in parallel. Effort must now be concentrated on treating the existing HCV patients, who are suffering from the failed treatments initiatives of the past.

The Egyptian Ministry of Health must take a two-pronged approach to tackling this disease. Currently, there are established DAAs that have the portent to eliminate HCV. In addition, local pharmaceutical companies should be aided in developing drug generic versions in order to make direct acting antiviral drugs more accessible for those who have not been able to gain access to treatment through governmentally subsidized schemes.

It is important to realize, however, that widespread DAA treatment will not solve all the associated problems of the HCV epidemic in Egypt. The complications of decompensated cirrhosis and hepatocellular carcinoma present a massive burden to Egyptian society and they still need to be addressed with adequate health care resource allocation.

Egypt has all the potential tools to tackle its HCV crisis. What is needed is ring-fenced governmental funding to enforce treatment policy, as well as clear medical and public health guidance in order to target treatment effectively. At the same time, the Egyptian Ministry of Health must continue to invest in research and development for the best emerging treatments to be implemented. However, it is important not to forget the essentials of education, infection control, equipment sterilization, and risk aversion to stop further growth of those who continue to become newly infected.
